# Alteration of urinary neutrophil gelatinase–associated lipocalin as a predictor of tacrolimus-induced chronic renal allograft fibrosis in tacrolimus dose adjustments following kidney transplantation

**DOI:** 10.1371/journal.pone.0209708

**Published:** 2018-12-21

**Authors:** Wiwat Chancharoenthana, Asada Leelahavanichkul, Salin Wattanatorn, Yingyos Avihingsanon, Kearkiat Praditpornsilpa, Somchai Eiam-Ong, Natavudh Townamchai

**Affiliations:** 1 Division of Nephrology, Department of Medicine, Chulalongkorn University, Bangkok, Thailand; 2 Excellent Center of Organ Transplantation (ECOT), King Chulalongkorn Memorial Hospital, Thai Red Cross Society, Bangkok, Thailand; 3 Immunology Unit, Department of Microbiology, Chulalongkorn University, Bangkok, Thailand; University of Toledo, UNITED STATES

## Abstract

Despite tacrolimus (TAC) drug-level monitoring, TAC-induced chronic renal allograft fibrosis remains an important problem. This study investigated the potential of urinary neutrophil gelatinase–associated lipocalin (uNGAL) as a chronic renal allograft fibrosis biomarker in a two-phase study (proof of concept and cohort). In the proof of concept stage of the study, increased TAC-doses at 3 days after dose adjustment compared with the baseline were associated with elevated uNGAL (+ΔuNGAL) and urinary interleukin 18 (IL-18), but normal serum creatinine (SCr), despite the therapeutic trough levels of TAC. In the cohort study, the patients with elevated uNGAL post-recruitment in comparison with the baseline (+ΔuNGAL) was associated with the more severe renal allograft fibrosis from renal pathology of the protocol biopsy at 12 months post kidney transplantation (post-KT). A cut-off value of uNGAL ≥ 125.2 ng/mL during a 3, 6, 9 and 12 months post-KT was associated with a higher fibrosis score, with an area under the receiver operating characteristics curve of 0.80 (95% confidence interval [CI] 0.72 to 0.88, *p* < 0.0001) and a hazard ratio (HR) of 2.54 (95% CI 1.45 to 9.33; *p* < 0.001). We conclude that uNGAL is a sensitive biomarker of TAC induced subtle renal injury and TAC-induced chronic renal allograft fibrosis. We propose that uNGAL measurements, in addition to trough levels of TAC, should be used to predict TAC-induced chronic renal allograft fibrosis in the recipients of KT.

## Introduction

Calcineurin inhibitor (CNI)-based immunosuppressive therapy predisposes kidney transplantation (KT) recipients to chronic renal allograft fibrosis in renal allografts [1]. Studies have reported CNI-induced nephrotoxicity in 40–50% and 100% of KT recipients 2 and 10 years post-KT, respectively, based on surveillance (protocol) renal allograft biopsies [2–4]. CNI-induced nephrotoxicity finally leads to chronic renal allograft fibrosis, with irreversible allograft loss [5]. Although tacrolimus (TAC), a calcineurin inhibitor, is an effective immunosuppressive drug, it is associated with a reduction of the glomerular filtration rate (eGFR) and chronic renal allograft fibrosis, even within the achieved TAC therapeutic range [6]. The latter may be due to variations in the optimal TAC dose (TAC_dose_) in each recipient [7, 8]. Hence, CNI dose adjustment is necessary to balance CNI toxicity/fibrosis versus rejection [9]. To delay the progression of chronic renal allograft fibrosis, early detection and rapid interventions are mandatory. Unfortunately, the optimal CNI dose for individual recipients remains uncertain [10]. Thus, chronic renal allograft fibrosis may occur in KT recipients with TAC trough levels within the therapeutic range at all stages of follow-up. It is possible that recommended TAC trough levels are relatively high, especially for the Asian population, due to the high prevalence of CYP3A5 expressors polymorphisms [11, 12]. A histopathology assessment from a renal biopsy is currently the only gold standard method for the detection of CNI-induced chronic renal allograft fibrosis [13]. At present, a biopsy is conducted 1, 6 and 12 months post-KT to detect CNI-induced chronic renal allograft fibrosis. If recommended therapeutic levels are achieved, the TAC doses given in the period shortly after post-KT are usually continued. In contrast, recipients who receive increased TAC doses in this period show a tendency towards continuation of higher TAC doses.

Non-invasive chronic renal allograft fibrosis-detection would allow more frequent evaluations and earlier interventions. Neutrophil gelatinase–associated lipocalin (NGAL) is a more sensitive biomarker of acute kidney injury (AKI) than serum creatinine (SCr), a conventional AKI biomarker [14, 15]. NGAL is also a sensitive biomarker of CNI-induced nephrotoxicity in nephrotic syndrome [16] and liver transplantation [17]. Previous research demonstrated reduced plasma and urinary NGAL (uNGAL) in heart transplant recipients receiving CNI-free immunosuppressive therapy [18]. Although serum NGAL and uNGAL show similar diagnostic value for AKI-detection [15], uNGAL might be a more appropriate biomarker to determine subtle kidney injury, considering that urine is a direct renal excretory product and that NGAL is produced mainly by the distal tubule of the kidney [19].

The aim of the present study was to investigate the potential of uNGAL as a chronic renal allograft fibrosis biomarker in a two-phase study (the proof of concept and the cohort). The following hypotheses were tested: i) uNGAL would be associated with subtle kidney injury after TAC dose adjustment in the proof of concept study (cross-sectional analysis), and ii) uNGAL would have potential as a predictor of TAC-induced chronic renal allograft fibrosis in the 12 month-cohort study.

## Material and methods

The study protocol was approved by Chulalongkorn University Institutional Review Board, and all the participants provided written informed consent. The clinical and research activities were consistent with the Principles of the Declaration of Istanbul, as outlined in the Declaration of Istanbul on Organ Trafficking and Transplant Tourism.

### Study design and recipient selection

Samples from all KT recipients at King Chulalongkorn Memorial Hospital, Thailand during January 2011 through December 2016 were used. The exclusion criteria were second KT, positive pre-transplant donor specific antibodies, combined extra-renal transplantation and ABO-incompatible KT. Furthermore, KT recipients with allograft impairment by some other causes including infection, antibiotics and recipients with any renal replacement therapy were also excluded. All of the participants received induction with basiliximab and maintenance with TAC, mycophenolate mofetil (2 g/day) and corticosteroids. TAC at 0.1 mg/kg/day was started at the time of the operation. Target TAC trough levels were 8–10 ng/mL for the first 6 months and reduced to 5–8 ng/mL thereafter. Surveillance renal biopsies were scheduled for 3, 6 and 12 months post-KT.

**Fig 1:**
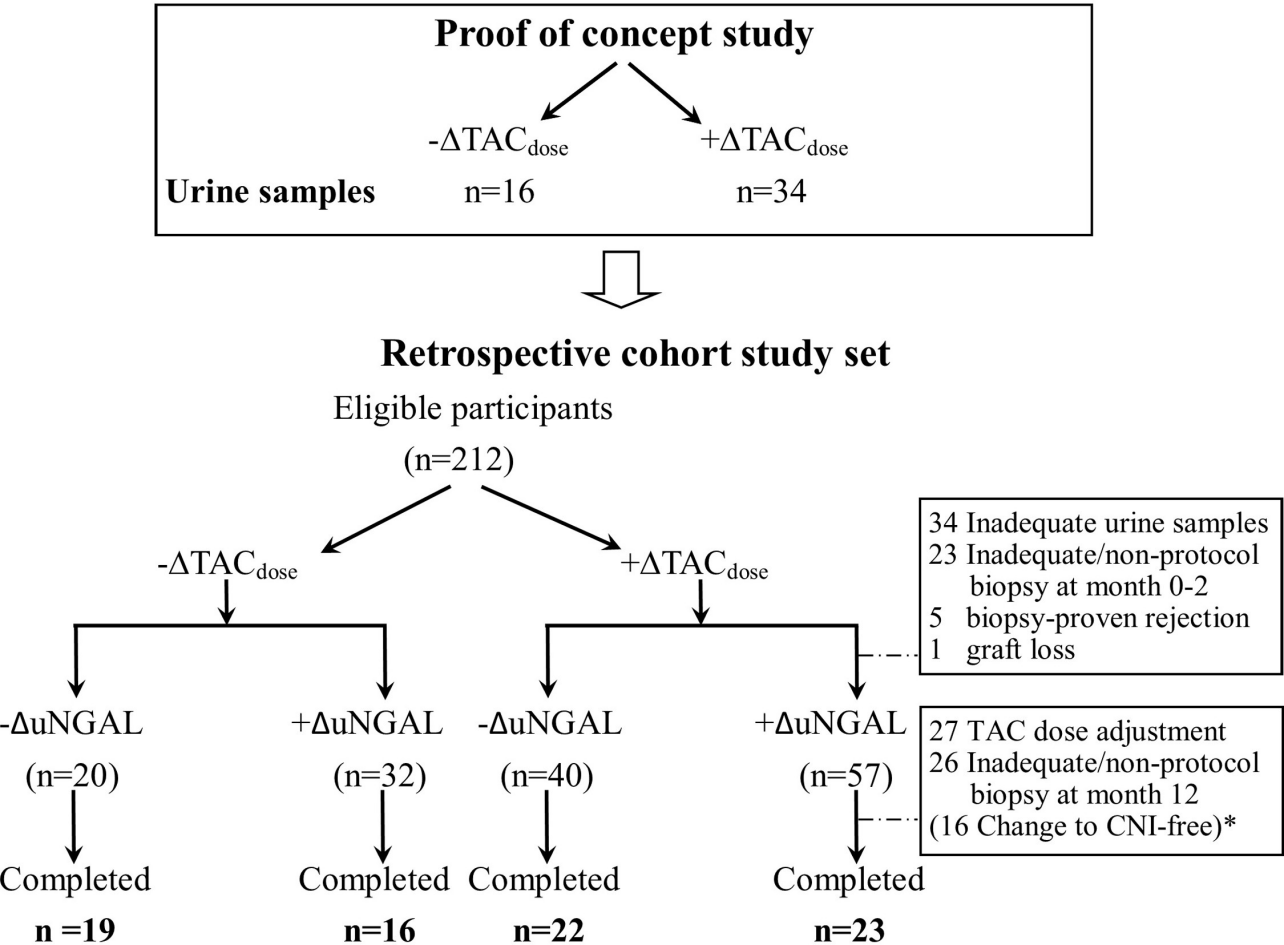
Characteristics of the recipients. CNI, calcineurin inhibitor; TAC, tacrolimus; uNGAL, urinary neutrophil gelatinase–associated lipocalin; Δ, change.

The study was conducted in two stages: a proof of concept and cohort phase **(Fig 1)**. The aim of the proof of concept phase of the study was to explore whether the alteration in absolute urinary NGAL (ΔuNGAL) was associated with kidney injury in a cross-sectional analysis of 50 recipients. In this phase, new KT recipients with less than 5% fibrosis on 3-month surveillance (protocol) renal biopsies were included within 120 days post-KT. Based on a comparison of TAC doses before and 3 days after TAC dose adjustment, these recipients were categorized as Day 0 and Day +3, respectively. They were then divided into two groups: those who received a decreased TAC_dose_ (-ΔTAC_dose_, *n* = 16) and those who received an increased TAC_dose_ (+ΔTAC_dose_, *n* = 34) and analysed with baseline ΔuNGAL values.

The aim of the cohort phase (*n* = 212) of the study was to determine whether the alteration in ΔuNGAL was associated with chronic renal allograft fibrosis at a 1-year follow-up. The inclusion criteria were all recipients with less than 5% fibrosis on surveillance renal biopsies who had no TAC dose adjustment for at least 12 months during the study. Samples were obtained for uNGAL and TAC trough levels within 4 weeks from the time of study enrolment and then at 3, 6, 9 and 12 months. One hundred thirty-two of 212 recipients were excluded for various reasons (**Fig 1**). Eighty recipients were divided into -ΔTAC_dose_ (*n* = 35) and +ΔTAC_dose_ groups (*n* = 45) based on the total dose adjustment (increased or decreased), TAC trough levels and uNGAL at baseline and after TAC dose adjustment.

Transplant renal biopsies were performed because of a protocol biopsy or clinical indications with 18-gauge core biopsy needles (BARD MAX-CORE, C. R. Bard, Inc., Tempe, AZ, USA). The adequacy of renal allograft samples was defined as biopsies containing at least seven glomeruli [20]. The renal allograft fibrosis data was semi-quantitatively scored by a pathologist blinded to the study based on Masson trichrome staining in accordance with the 2007 Banff classification [20]. Fibrosis relative to the total area was scaled as 0 to 3 (up to 5%, 6–25%, 26–50% and greater than 50%, respectively) in the renal interstitium (*ci*), tubules (*ct*) and vessels (*cv*) [20]. The primary study endpoint was fibrosis severity in allografts 12 months post-KT. The secondary endpoints were renal allograft survival, acute rejection rates (both antibody-mediated and cellular rejection) and systemic infections 24 months post-KT.

### Laboratory measurements

Urine samples were centrifuged at 3000x *g* for 10 min and kept at -70°C until used. uNGAL and urinary IL-18, IL-6 and tumour necrosis factor α (TNF-α) were measured by enzyme-linked immunosorbent assay kits (Raybiotech, Norcross, GA, USA for urinary IL-18 and R&D Systems, Minneapolis, MN, USA for the others). Serum creatinine (SCr), urinary creatinine (uCr), and 24-h urinary total protein were measured using an enzymatic-based biochemical analyser (Vitros 4600 Chemistry System, Ortho-Clinical Diagnostics, Rochester, NY, USA). The estimated glomerular filtration rate (eGFR) was calculated from SCr using the Modification of Diet in Renal Disease equation [21].

### Statistical analysis

Baseline characteristics of the recipients are presented as mean ± standard deviation (SD). Other data are presented as median ± interquartile ranges. The student’s *t*-test or Mann–Whitney *U* test and chi-squared or Fischer’s exact test were conducted to compare continuous variables and categorical variables, respectively. The associations between uNGAL, eGFR and other study endpoints were assessed with Cox proportional hazard regression analysis and performed an intention to treat analysis. An area under the curve (AUC) and receiver operator characteristic (ROC) analysis, with C-statistic-calculation (for discrimination accuracy) was performed [22]. STATA version 13.1 (StataCorp., College Station, TX, USA) was used for all analyses. A *p-*value < 0.05 was considered a statistically significant difference.

## Results

### Kidney injury after TAC dose adjustment detected by uNGAL: a cross-sectional analysis

**Fig 2:**
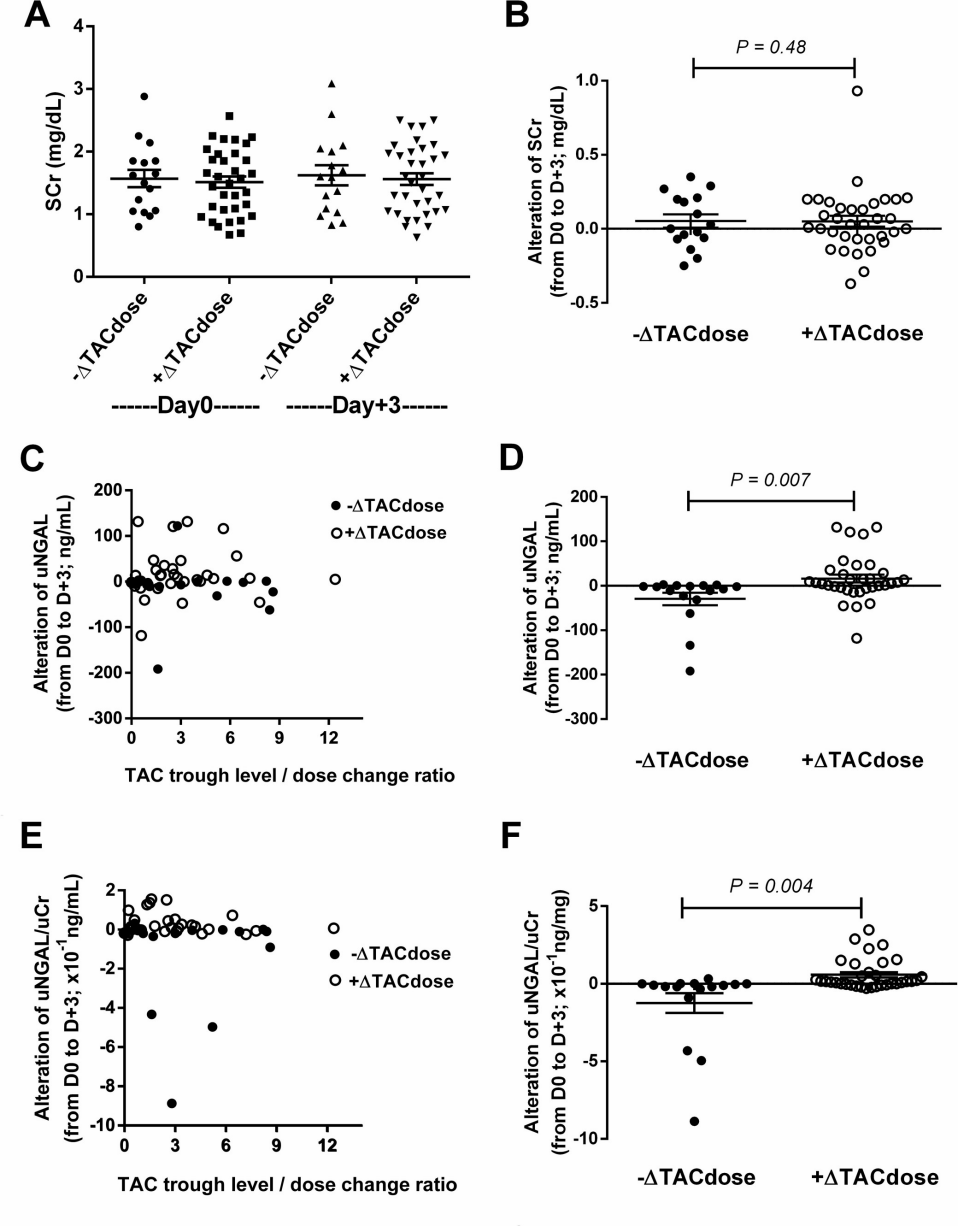
Comparison of renal injury markers before (D 0) and 3 days after (D +3) TAC dose adjustment in the -ΔTAC_dose_ and +ΔTAC_dose_ groups. SCr and the alteration in SCr from the baseline were comparable between recipients in the decreased TAC dose (-ΔTAC_dose_) and increased TAC dose (+ΔTAC_dose_) groups **(A** and **B)**. There were no differences in uNGAL and uNGAL/uCr between the -ΔTAC_dose_ and +ΔTAC_dose_ groups **(C, E)** but the alteration in absolute uNGAL (ΔuNGAL) and uNGAL/uCr from the baseline were higher in the +ΔTAC_dose_ group **(D, F)**.

**Fig 3:**
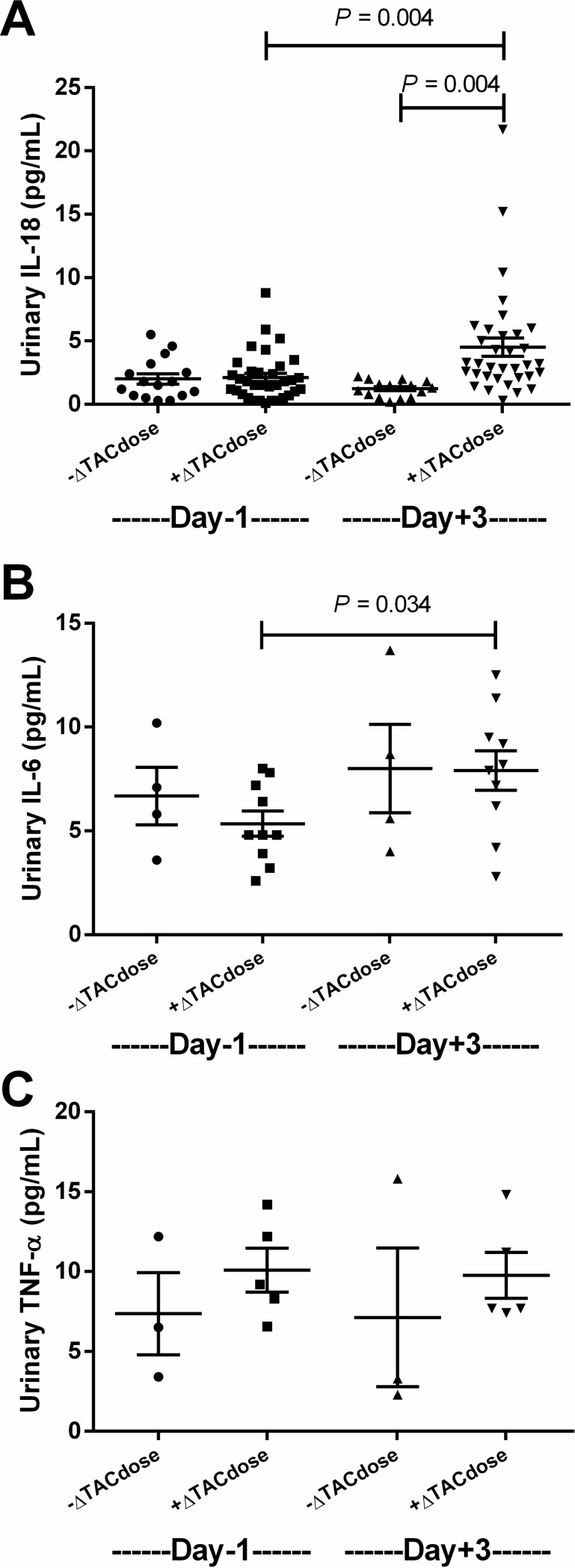
Comparison of urinary cytokines between the day before (D 0) and 3 days after (D +3) TAC dose adjustment in the -ΔTAC_dose_ and +ΔTAC_dose_ groups. Both urinary IL-18 **(A)** and IL-6 **(B)** but not TNF-α were higher in recipients with increased TAC doses after dose adjustment.

Most of the KTs in the cross-sectional analysis were living-related donor transplantation recipients (*n* = 37, 74%) (**Fig 1**). At the time of enrolment, the median time since the KT was 6.5 months (1.5–11 months). The baseline eGFR was 93.4 (79.4–110.9) mL/min/1.73m^2^. According to prescribed TAC doses on Day +3 versus Day 0, 16 and 34 recipients were classified as -ΔTAC_dose_ (decreased dose) and +ΔTAC_dose_ (increased dose), respectively. The mean difference in TAC trough levels between Day +3 and Day 0 was –2.40 ± 0.8 (-0.3 to -8.4) and 2.37 ± 0.6 (0.01 to 17.4) ng/mL in the -ΔTAC_dose_ and +ΔTAC_dose_ group, respectively. Interestingly, SCr, uNGAL and uNGAL/uCr on Day +3 were not different as compared with those on Day 0 of TAC dose adjustment **(Fig 2A, 2C and 2E).** Furthermore, SCr values alteration was unchanged in the -ΔTAC_dose_ and +ΔTAC_dose_ groups on Day +3 versus Day 0 **(Fig 2B)**. However, the alteration in ΔuNGAL and uNGAL/uCr values were higher in the +ΔTAC_dose_ group than -ΔTAC_dose_ group **(Fig 2D and 2F)**. In addition, urinary IL-18 and IL-6 but not urinary TNF-α were increased on Day +3 as compared with Day 0 in the +ΔTAC_dose_ group **(Fig 3),** pointing to renal injury after TAC dose adjustment.

### Renal allograft fibrosis and renal allograft function in recipients with increased uNGAL (+ΔuNGAL) in a longitudinal 1-year follow-up

**Fig 4:**
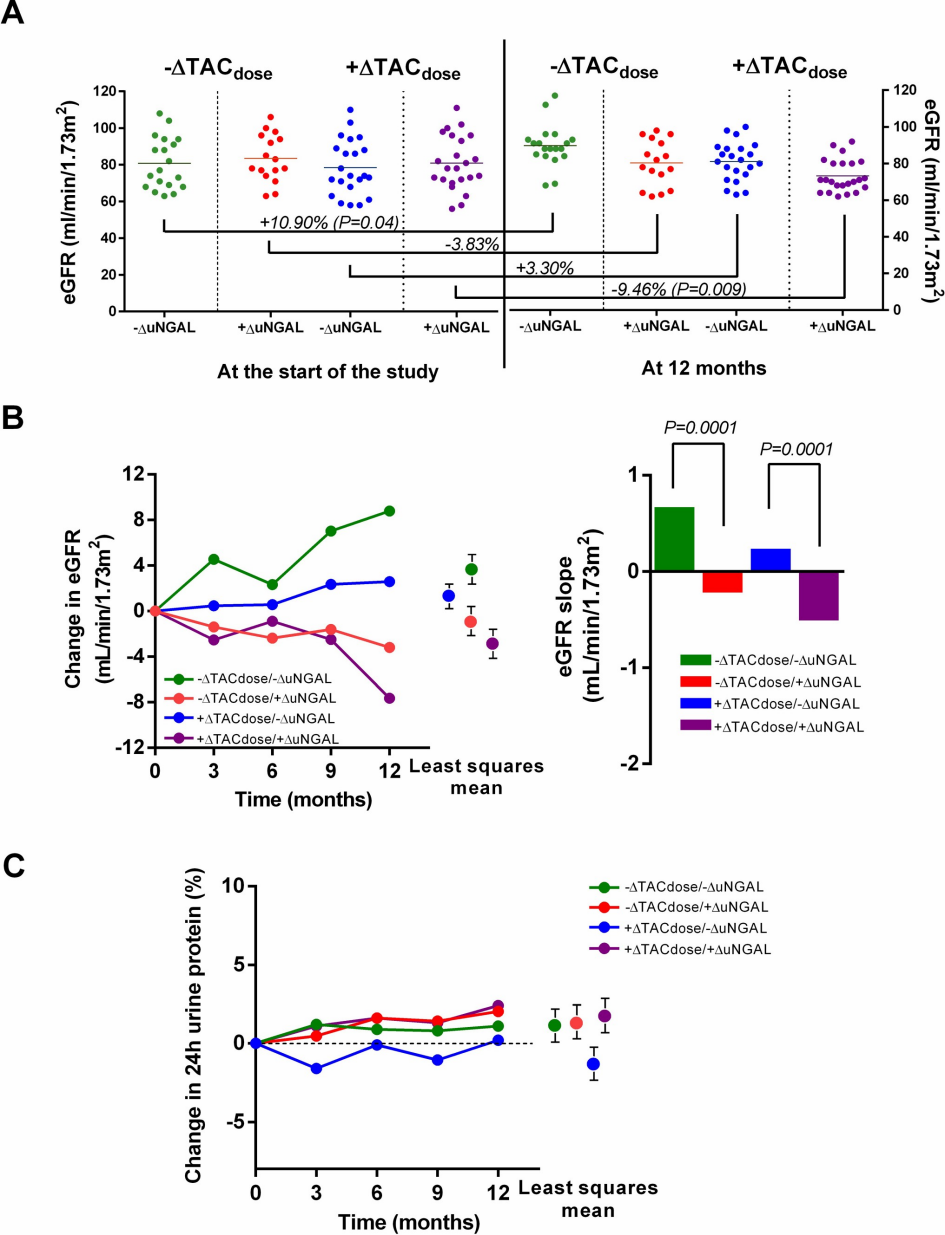
Renal allograft histology, change in eGFR and percentage change in 24-h proteinuria during the study period. A scatter plot showing eGFR and the percentage alteration in mean values of eGFR in the -ΔTAC_dose_ and +ΔTAC_dose_ groups at enrolment versus the values observed 12 months post-enrolment **(A)**. The time course of changes in eGFR in all four subgroups **(B, left side) (B, right side**), in addition to the percentage change in 24-h urinary protein, in each group **(C)** *(*p* < 0.01), ***(*p* < 0.001).

The demographic data are presented in **Table 1**. The recipients were divided into different groups according to the ΔTAC_dose_ (increased/decreased) and ΔuNGAL values **(Fig 4A)**. Mean TAC trough levels showed no statistically significant difference between -ΔTAC_dose_ and +ΔTAC_dose_ group in every 3-month follow-up with also no difference at 12-month (6.8 ± 0.9 vs. 7.1 ± 1.1 ng/mL, respectively) **(S1 Table)**. Unadjusted eGFR at the time of enrolment was 80.9 ± 14.1, 83.5 ± 12.9, 78.5 ± 15.1 and 80.9 ±1 4.7 mL/min/1.73m^2^ in the -ΔTAC_dose_/-ΔuNGAL, -ΔTAC_dose_/+ΔuNGAL, +ΔTAC_dose_/-ΔuNGAL and +ΔTAC_dose_/+ΔuNGAL groups, respectively **(Fig 4A)**. At the 12-month observation, only the eGFR in the +ΔTAC_dose_/+ΔuNGAL group was lower than the base-line (80.9 ± 14.7 vs. 73.3 ± 8.8 mL/min/1.73 m^2^). Despite the reduced TAC_dose_ in the -ΔTAC_dose_ group, the eGFR progressively declined in cases where the uNGAL increased from baseline (-ΔTAC_dose_/+ΔuNGAL vs. -ΔTAC_dose_/-ΔuNGAL; *p* < 0.001), as shown in **Fig 4A**. Increased uNGAL predicted worsening renal function in the -ΔTAC_dose_ group. The annual slope of eGFR decline was 0.2 and 0.5 mL/min/1.73m^2^ per year in the -ΔTAC_dose_/+ΔuNGAL and +ΔTAC_dose_/+ΔuNGAL group, respectively. On the other hand, the annual slope of eGFR increase was 0.7 and 0.2 mL/min/1.73m^2^ per year in the -ΔTAC_dose_/-ΔuNGAL and +ΔTAC_dose_/-ΔuNGAL groups, respectively (**Fig 4B**). A repeated measures analysis showed a least squares mean change in the eGFR (mL/min/1.73m^2)^ (95% confidence interval; CI) from the baseline in the +ΔTAC_dose_/+ΔuNGAL, -ΔTAC_dose_/-ΔuNGAL, -ΔTAC_dose_/+ΔuNGAL and +ΔTAC_dose_/-ΔuNGAL groups of -3.3 (-0.2 to -4.2), 3.8 (2.7 to 5.5), -0.7 (–0.1 to –2.4) and 0.5 (0.1 to 3.2), respectively (**Fig 4B**). Nevertheless, 24-h urinary protein remained stable in all the groups, with a trend towards an increase in the +ΔuNGAL group (**Fig 4C**). The distribution of the relative change in the Banff score was highest in the +ΔTAC_dose_/+ΔuNGAL group, followed by the -ΔTAC_dose_/+ΔuNGAL, +ΔTAC_dose_/-ΔuNGAL and -ΔTAC_dose_/-ΔuNGAL groups. Of note, in the -ΔTAC_dose_ group, recipients who had +ΔuNGAL had more increased chronic characteristics of Banff score at 12 months compared with those had -ΔuNGAL, particularly in arteriolar hyalinosis **(Table 2)**. Moreover, recipients in the +ΔTAC_dose_/+ΔuNGAL group demonstrated an earlier presentation of chronic characteristics Banff score (at 6 months) compared with recipients in the +ΔTAC_dose_/-ΔuNGAL group. These findings point to a role for ΔuNGAL as a predictor of chronic renal allograft fibrosis.

**Table 1:** Baseline demographics.

Characteristics	Proof of concept study				Cohort study						
	All recipients (N = 50)	-ΔTAC_dose_ (N = 16)	+ΔTAC_dose_ (N = 34)	*p*-value	All recipients (N = 80)	-ΔTAC_dose_		*p*-value	+ΔTAC_dose_		*p*-value
						-ΔuNGAL (n = 19)	+ΔuNGAL (n = 16)		-ΔuNGAL (n = 22)	+ΔuNGAL (n = 23)	
Age (years), mean±SD	48.4±9.2	45.6±8.6	49.4±7.4	0.11	52.4±14.2	48.3±11.5	52.8±14.1	0.31	49.0±10.3	50.2±11.9	0.72
Sex (male), n (%)	34 (68.0)	10 (58.8)	24 (70.6)	0.61	55 (68.8)	16 (84.2)	13 (81.3)	0.82	12 (54.5)	14 (60.9)	0.89
Types of donor, living, n (%)	36 (72.0)	11 (68.8)	25 (73.5)	0.99	45 (56.3)	12 (63.2)	9 (56.3)	0.95	10 (45.5)	16 (69.6)	0.18
Donor age (years), mean±SD	44.8±8.5	43.6±6.9	45.6±8.1	0.40	51.1±15.1	49.6±11.3	43.8±13.5	0.18	46.1±11.6	49.3±13.6	0.40
Cold ischemic time, h, mean±SD	17.2±6.4	16.6±6.8	17.7±9.9	0.69	20.1±9.1	17.6±8.8	18.6±6.9	0.71	16.4±7.7	18.1±6.0	0.41
Follow-up* (months), mean±SD	3.5±0.2	3.6±0.1	3.5±0.2	0.07	11.6±5.2	13.4±6.6	10.5±2.8	0.22	12.8±4.2	11.3±6.8	0.38
Causes of ESRD, n (%)											
Diabetes	26 (52.0)	10 (62.5)	16 (47.1)	0.48	31 (38.8)	11 (57.9)	7 (43.8)	0.62	4 (18.2)	9 (39.1)	0.22
Glomerulonephritis	10 (20.0)	6 (37.5)	4 (11.8)	0.08	19 (23.8)	2 (10.5)	6 (37.5)	0.14	7 (31.8)	4 (17.4)	0.44
ADPKD	0 (0)	0 (0)	0 (0)	-	3 (3.7)	1 (5.3)	2 (12.5)	0.88	0 (0)	0 (0)	-
Obstructive uropathy	0 (0)	0 (0)	0 (0)	-	1 (1.2)	0 (0)	1 (6.3)	0.92	0 (0)	0 (0)	-
Unknown	14 (28.0)	5 (31.3)	9 (26.5)	0.99	26 (32.5)	9 (47.4)	6 (37.5)	0.81	4 (18.2)	7 (30.4)	0.55
PRA, %, mean±SD	8.3±11.5	7.7±10.9	8.8±12.3	0.76	12.6±25.4	7.9±22.6	8.9±20.4	0.89	10.4±27.3	9.5±21.5	0.90
HLA mismatch, mean±SD	1.8±0.6	2.3±1.1	1.9±0.7	0.13	3.6±1.1	4.1±1.9	3.8±1.9	0.64	3.4±1.7	3.9±1.5	0.30
Induction, n (%)	50 (100)	16 (100)	34 (100)	-	80 (100)	19 (100)	16 (100)	-	22 (100)	23 (100)	-
Medications, n (%)											
ACE inhibitor	0 (0)	0 (0)	0 (0)	-	24 (30.0)	5 (26.3)	6 (37.5)	0.73	9 (40.9)	4 (17.4)	0.16
ARB	8 (16.0)	3 (18.8)	5 (14.7)	0.96	38 (47.5)	9 (47.4)	12 (75.0)	0.19	7 (31.8)	10 (43.5)	0.62
Beta-blockers	3 (6.0)	2 (12.5)	1 (2.94)	0.49	27 (33.8)	5 (26.3)	7 (43.8)	0.47	6 (27.3)	9 (39.1)	0.60
CCB non-dihydropyridine	0 (0)	0 (0)	0 (0)	-	3 (3.8)	2 (10.5)	1 (6.3)	0.87	0 (0)	0 (0)	-
CCB dihydropyridine	11 (22.0)	4 (25.0)	7 (20.1)	0.98	26 (32.5)	6 (31.6)	7 (43.8)	0.69	4 (18.2)	9 (39.1)	0.22
ACE inhibitor and ARB	0 (0)	0 (0)	0 (0)	-	12 (15.0)	4 (21.1)	3 (18.8)	0.80	2 (9.1)	3 (13.0)	0.95

ACE, angiotensin converting enzyme; ADPKD, autosomal dominant polycystic kidney disease; ARB, angiotensin II receptor blocker; CCB, calcium channel blocker; HLA, human leukocyte antigen; PRA, panel reactive antibody; ESRD, end-stage renal disease; SD, standard deviation; uNGAL, urinary neutrophil gelatinase-associated lipocalin; Δ, change.

*posttransplant follow-up time prior to study period.

**Table 2:** Changes in chronic biopsy scores in kidney allografts with time post-KT.

Time (post-KT; months)	Banff score (in chronic feature domains)				
	*Ah*^†^	*cg*^†^	*ci*^†^	*ct*^†^	*cv*^†^
	**-ΔTAC**_**dose**_/**-ΔuNGAL** (n = 19)				
3	0.10±0.21	0.00±0.00	0.01±0.08	0.21±0.44	0.01±0.05
6	0.13±0.54	0.00±0.00	0.01±0.09	0.32±0.57	0.08±0.16
12	0.21±0.53	0.01±0.01	0.36±0.75**	0.66±0.74**	0.12±0.82
	**-ΔTAC**_**dose**_/+**ΔuNGAL** (n = 16)				
3	0.09±0.36	0.00±0.00	0.06±0.12	0.15±0.07	0.01±0.03
6	0.16±0.57	0.01±0.01	0.33±0.41*	0.62±0.48*	0.55±0.36*
12	0.82±0.89**	0.03±0.02	0.84±0.69**	0.95±0.80**	0.68±0.98
	**+ΔTAC**_**dose**_/**-ΔuNGAL** (n = 22)				
3	0.10±0.08	0.00±0.00	0.10±0.50	0.33±0.43	0.00±0.00
6	0.24±0.73	0.01±0.23	0.22±0.59	0.88±0.69*	0.61±0.87*
12	0.75±0.96**	0.02±0.01	0.96±0.83**	1.03±0.38	0.55±0.65
	**+ΔTAC**_**dose**_/**+ΔuNGAL** (n = 23)				
3	0.04±0.09	0.00±0.00	0.08±0.03	0.26±0.54	0.00±0.00
6	0.90±0.84*	0.02±0.69	0.44±0.63*	0.89±0.38*	0.31±0.49*
12	1.17±0.93**	0.03±0.20	0.84±0.51**	1.45±0.66**	0.60±0.78**

For this analysis the scores of individuals’ graft at different points of time post-KT were compared with themselves by paired analysis.

*Significantly different from 3-month value.

**Significantly different from 6-month value. All statistics done by non-parametric paired *t*-test.

^†^*ah*, arteriolar hyalinosis; *cg*, chronic glomerulopathy; *ci*, interstitial fibrosis; *ct*, tubular atrophy; *cv*, chronic vasculopathy.

**Fig 5:**
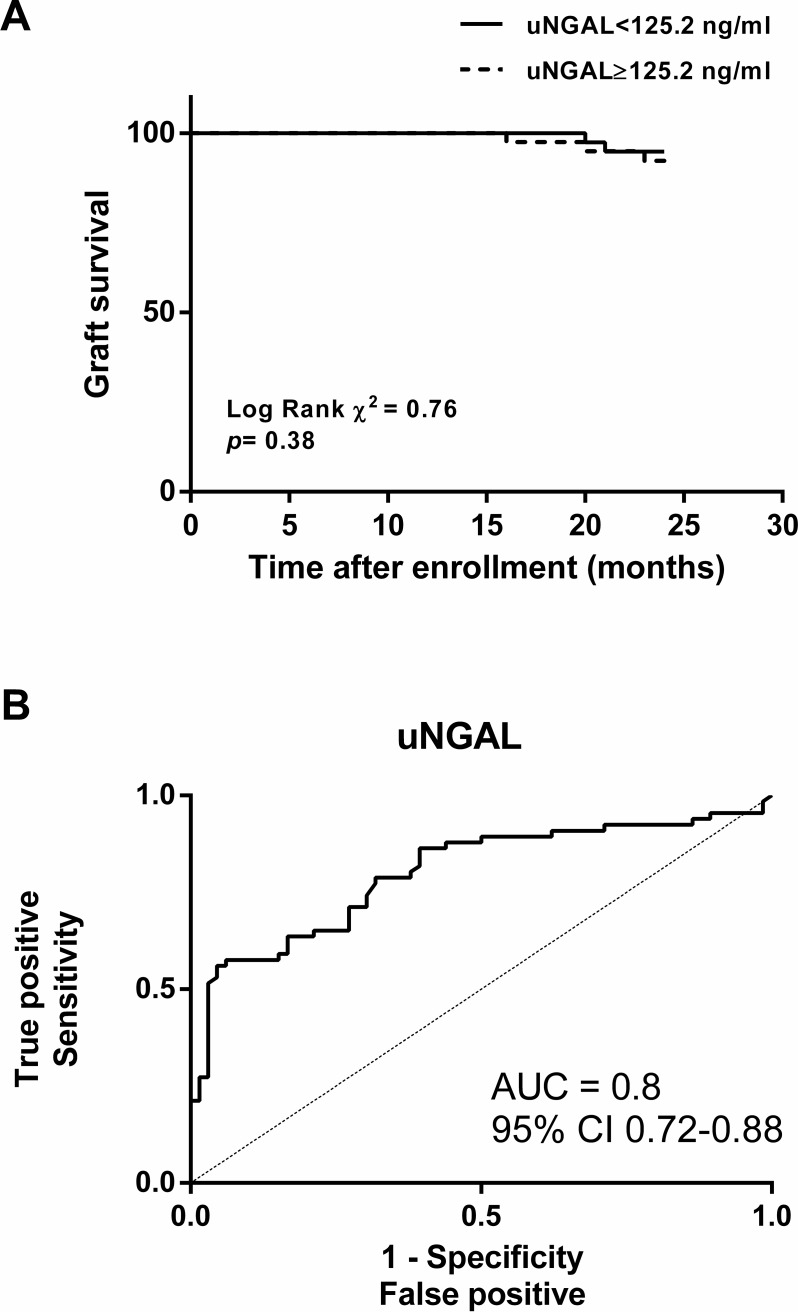
Urinary NGAL was predictive of chronic renal allograft fibrosis at 12 months post-enrolment. Kaplan–Meier curve for the association of chronic renal allograft fibrosis with uNGAL **(A)** and ROC curve for the association between uNGAL at enrolment and chronic renal allograft fibrosis **(B)** were demonstrated.

As the optimal cut-point of uNGAL in the cohort was 125.2 ng/mL, this value was used for the graft survival analysis. Graft survival 2 years post-enrolment of recipients with uNGAL < 125.2 and ≥ 125.2 ng/mL was 94.9% and 89.5%, respectively (*p* = 0.38), as shown in **Fig 5A**. The most common causes of graft loss were chronic antibody-mediated rejection (CAMR) (67%) and recurrent glomerular diseases (33%). There was a zero incidence of acute cellular rejection and BK infection. It is to be noted that both events developed after 1-year follow-up which supports an evidence of chronic renal allograft fibrosis resulting from TAC rather than other specific etiologies. The AUC ROC of uNGAL for chronic renal allograft fibrosis was 0.80 (95% confidence interval [CI] 0.72 to 0.88, *p* < 0.0001) (**Fig 5B**) with a sensitivity of 82% and a specificity of 88%. In a multivariate model, including donor and recipient age, baseline SCr, and uNGAL levels, the association of uNGAL ≥ 125.2 ng/mL with chronic renal allograft fibrosis in the 12-month biopsy remained **(Table 3)**. In addition, long-term chronic renal allograft fibrosis was associated with high baseline SCr (over 1.5 mg/dL, *p* = 0.01) and an increased TAC_dose_ (*p* = 0.02) but not with donor and recipient age.

**Table 3:** Determinants of renal allograft interstitial fibrosis and tubular atrophy at the time of enrolment using univariate and multivariate Cox proportional hazards model.

Characteristics	Crude HR (95% CI)	*P-*value	Adjusted HR (95% CI)	*P-*value
Donor age	0.88 (0.81 to 0.99)	**0.03**	0.96 (0.85 to 1.1)	0.14
Recipient age	1.06 (0.55 to 1.63)	0.15	1.1 (0.95 to 1.17)	0.99
Serum creatinine ≥1.5 *vs*. <1.5 mg/dL^a^	1.22 (0.71 to 2.21)	0.06	2.07 (0.79 to 5.19)	**0.01**
ΔTAC_dose_ increase *vs*. decrease	3.6 (2.7 to 6.33)	**<0.001**	2.59 (1.66 to 5.31)	**0.02**
uNGAL ≥125.2 ng/mL *vs*. <125.2 ng/mL^b^	2.11 (1.4 to 8.22)	**<0.001**	2.54 (1.45 to 9.33)	**<0.001**

^a^Serum creatinine at the enrolment ≥1.5 *vs*. <1.5 mg/dL and

^b^uNGAL ≥125.2 ng/mL *vs*. <125.2 ng/mL correspond to the median of the distribution of the creatine and uNGAL values in the cohort, respectively.

To test the potential of uNGAL as a chronic renal allograft fibrosis biomarker, uNGAL was added to a model with follow-up period and conventional risk factors (i.e. age [donors and recipients], sex [donors], numbers of anti-hypertensive medications at baseline and ΔTAC_dose_). The addition of uNGAL increased the *c* statistic to 0.84 (95% CI, 0.78 to 0.90).

## Discussion

The present study demonstrated a benefit of uNGAL measurements, in combination with TAC trough level monitoring, in KT. An increase in uNGAL from the baseline was associated with renal allograft injury following TAC dose adjustment, despite achievement of target TAC trough levels. KT recipients who had a trend of the higher uNGAL as compared with the baseline at 1-year follow-up showed the more severe fibrosis than the recipients with the lower uNGAL. In addition, the present study demonstrated that a decreased TAC_dose_ improved the eGFR and decreased renal allograft fibrosis. In the cross-sectional arm of the study, we demonstrated an uncertain correlation between TAC dose adjustment and TAC trough level, suggesting that variability in TAC responses in individual recipients might enhance renal allograft fibrosis progression [23]. Interestingly, the increased doses of TAC resulted in the elevation of uNGAL, uNGAL/uCr (from the baseline) and urinary cytokines but not enhanced SCr. This finding implies that alterations in uNGAL and the absolute values urinary cytokine are very sensitive markers of a subtle TAC-induced kidney injury. Previous studies showed that uNGAL was elevated in KT recipients with renal allograft injury, delayed graft function and long-term renal allograft function, as well as in liver-transplant recipients with CNI toxicity [16, 24, 25]. In the present study, the absence of any difference in chronic renal allograft fibrosis discrimination between uNGAL and uNGAL/uCr implies that uNGAL could serve as a marker of chronic renal allograft fibrosis, independent of urinary concentration level [26, 27]. Hence, we used the alteration in absolute uNGAL (ΔuNGAL) as the main determinant biomarker of subtle renal injury in the cohort study.

In the cohort study, there were no differences among the groups at the time of enrolment in SCr, fibrosis score and the established renal allograft fibrosis risk factors [28–30] (reno-protective renin-angiotensin-aldosterone system blocking agents [31, 32]). At the 12-month post-recruitment, the prevalence of chronic renal allograft fibrosis was higher in recipients whose TAC doses were increased in the early phase of post-KT, and such doses were continued because of the achieving of the TAC trough levels. Although this practice complies with current guidelines [9], the aforementioned finding implies that i) the current recommended TAC trough level might be too high, especially in an Asian population and ii) a non-invasive biomarker of chronic renal allograft fibrosis, together with TAC level monitoring, is needed. Interestingly, the renal biopsy 6 months post-KT did not show CNI toxicity or chronic renal allograft fibrosis in both recipients with increased and decreased TAC doses from the baseline. However, the renal biopsy 12 months post-recruitment revealed more chronic renal allograft fibrosis in recipients with increased TAC doses. These findings suggest that the diagnosis of CNI toxicity by a renal biopsy may not be sensitive enough and that other biomarkers are needed.

Interestingly, both ΔuNGAL (compared with the baseline) and the fibrosis score were higher at the 12-month follow-up in those with increased TAC doses as compared with the baseline at the time of enrolment (+ΔTAC_dose_), indicating that a trend of ΔuNGAL may be a good predictive biomarker for TAC-induced chronic renal allograft fibrosis rather than a single time measurement. This postulation is also supported by the unchanged or decreased uNGAL between Day 0 and Day +3 despite reduced TAC dose in the proof of concept part of our study (**Fig 2C and 2D**). Likewise, in the cohort study, there is a group with increased uNGAL after the reduction of TAC dose. Indeed, uNGAL was also strongly associated with renal fibrosis in primary glomerulonephritis [33], acute rejection and immune-related renal allograft fibrosis [34]. Although the leading cause of graft loss in our cohort is due to CAMR and recurrent glomerulonephritis, the serial protocol biopsy could preclude an immune-related CNI toxicity. Moreover, ΔuNGAL was associated with renal allograft eGFR at 12 months post-KT but not 6 months post-KT. This suggests a cumulative effect of subclinical allograft injury that once an event has occurred, the interventions at the late post-KT shows a little effect [35]. In addition, the fibrosis score was less severe in recipients with a decreased TAC dose from the baseline (-ΔTAC_dose_) compared with the +ΔTAC_dose_ group (*p* < 0.05). The increase in uNGAL levels of recipients, in the present study, in the -ΔTAC_dose_ group indicates possible renal allograft fibrosis progression.

In the present study, we observed the greatest decline of adjusted eGFR at 12 months post-enrolment in the recipients with an increased-TAC dose plus increased-uNGAL from the baseline (+ΔTAC_dose_/+ΔuNGAL). Indeed, renal allograft function in recipients with a decreased TAC dose (-ΔTAC_dose_) was better than that of recipients with increased doses (+ΔTAC_dose_). Recipients with a decreased-TAC dose from the baseline and decreased-uNGAL (-ΔTAC_dose_/-ΔuNGAL) showed the lowest kidney injury progression. In addition, there was a trend towards higher 24-h proteinuria in the +ΔTAC_dose_/+ΔuNGAL group as compared with the +ΔTAC_dose_/-ΔuNGAL groups at the 12-month post-enrolment, supporting an association between i) increased uNGAL (+ΔuNGAL) and chronic renal allograft fibrosis and ii) high proteinuria and chronic renal allograft fibrosis [36]. In the present study, proteinuria among KT recipients was a predictive marker of long-term renal allograft survival [36, 37]. Increased 24-h urinary protein in the early stages after transplantation not only indicated enhanced renal allograft fibrosis progression but also rapid deterioration of allograft function, particularly in the +ΔTAC_dose_/+ΔuNGAL group (**Fig 4B and 4C**). This finding implies that ΔuNGAL might be a more sensitive, or at least non-inferior, biomarker than proteinuria as a predictor of renal allograft fibrosis progression. And uNGAL ≥ 125.2 ng/mL (AUC ROC at 0.80) was an additional predictor of chronic renal allograft fibrosis in our cohort. The increased C statistic (AUC ROC of 0.84) implies that the addition of uNGAL measurements to TAC monitoring (trough levels and dosing trends) improved the concordance between predicted and observed chronic renal allograft fibrosis. Some studies have demonstrated an elevated ΔuNGAL in recipients with various settings [38, 39]; therefore, we subsequently evaluated ΔuNGAL from those with acute antibody mediated rejection, acute cellular rejection, chronic antibody-mediated rejection (CAMR), and normal allograft function as the control groups **(Table 4)**. Although ΔuNGAL in among recipients with kidney injury were higher than those with normal allograft function, recipients with either CAMR or TAC-induced chronic renal allograft fibrosis showed robust ΔuNGAL compared with those with acute rejection. This attributed to uNGAL an interesting predictive value of renal allograft fibrosis; however, the numbers in among groups are small. Accordingly, in our opinion, uNGAL measurement should be performed and interpreted with a fair tentative diagnosis by taking a careful history, utilizing and combining available immunological diagnostic testing.

**Table 4:** Median urinary NGAL levels stratified by status of renal allograft function.

Renal allograft status (biopsy-proven)	uNGAL (ng/mL)^a^	*P-*value ^b^
Normal renal allograft function (n = 32)	26.7 (0.0 to 79.8)	-
Acute cellular rejection (n = 3)	228.3 (74.5 to 450.3)	**<0.01**
Acute antibody mediated rejection (n = 4)	311.2 (64.8 to 922.6)	**<0.01**
Chronic active antibody mediated rejection (n = 8)	793.6 (192.7 to 3,566.3)	**<0.001**
TAC-induced chronic renal allograft fibrosis (n = 17)	522.1 (148.4 to 1,828.9)	**<0.001**

^a^Median urinary NGAL (ng/mL) level were stratified according to renal allograft status determined by biopsy-proven diagnosis

^b^ΔuNGAL defines as the difference between baseline (from stored urine) and time of diagnosis. The *P*-values were determined by using the Kruskal–Wallis test.

There were several limitations in our study. First, this was a retrospective cohort study and therefore cannot accurately demonstrate causal-inference relationships, despite the use of matched-pair renal allograft biopsies. Second, for minimizing the potential intervening cause of immune-related renal allograft fibrosis, the current study excluded high immunological risk KT recipients as described in the Material and Methods section. Therefore, the results cannot be extrapolated to all KT recipients. Third, most of the recipients were in the first year post-KT and had early-stage renal allograft fibrosis. Further studies with longer follow-up periods are needed.

In conclusion, based on the findings of the present study, we propose that uNGAL measurements, together with TAC trough level monitoring, may predict TAC-induced chronic renal allograft fibrosis in KT recipients. In the present study, chronic renal allograft fibrosis was detected in renal allografts, despite recipients achieving TAC therapeutic levels. Thus, a non-invasive fibrosis biomarker should be helpful for early renal preservation procedures. In addition to TAC trough levels, uNGAL should be frequently evaluated in routine KT follow-up. Further studies are warranted.

## Supporting information

S1 File Data_average change of uNGAL–uCr. (XLSX)

S1 Table Comparison of mean TAC trough levels between -ΔTAC_dose_ and +ΔTAC_dose_ group during follow-up. (DOCX)

## Abbreviations

ACE: angiotensin converting enzyme
AKI: acute kidney injury
AUC: area under the curve
*ci*_x_: chronic interstitium grade *x*
*ct*_x_: chronic tubule grade *x*
*cv*_x_: chronic vascular grade *x*
CAMR: chronic antibody-mediated rejection
CCBs: calcium channel blockers
CNI: calcineurin inhibitor
eGFR: estimated glomerular filtration rate
ESRD: end-stage renal disease
HLA: human leukocyte antigen
HR: hazard ratio
IDI: integrated discrimination improvement
IL-6: interleukin-6
IL-18: interleukin 18
KT: kidney transplantation
NRI: net reclassification improvement
ROC: receiver operator characteristic
SD: standard deviation
SCr: serum creatinine
TAC: tacrolimus
TNF-α: tumour necrosis factor α
uCr: urinary creatinine
uNGAL: urinary neutrophil gelatinase–associated lipocalin
