# Correction: Alteration of urinary neutrophil gelatinase-associated lipocalin as a predictor of tacrolimus-induced chronic renal allograft fibrosis in tacrolimus dose adjustments following kidney transplantation

**DOI:** 10.1371/journal.pone.0213009

**Published:** 2019-02-22

**Authors:** The PLOS ONE Staff

The first two authors, Wiwat Chancharoenthana and Asada Leelahavanichkul, should be noted as equal co-corresponding authors.

There is an error in the Funding statement. The correct Funding statement is as follows: All of the funding supports during study period including the Development of New Faculty Staff fund and Ratchadapiseksomphot Endowment Fund 2017 (76001-HR), Faculty of Medicine, Chulalongkorn University and National Science and Technology Development Agency (NSTDA: P-13-00505)–Dr. Asada Leelahavanichkul. A.L. is under Center of Excellence in Immunology and Immune Mediated Diseases group. The funders had no role in study design, data collection and analysis, decision to publish, or preparation of the manuscript.

The publisher apologizes for the errors.
