# Supplementary material for: Alteration of urinary neutrophil gelatinase–associated lipocalin as a predictor of tacrolimus-induced chronic renal allograft fibrosis in tacrolimus dose adjustments following kidney transplantation
Source: PLoS One. 2018 Dec 21;13(12):e0209708. doi: 10.1371/journal.pone.0209708 (PMC6303063; doi:10.1371/journal.pone.0209708)
Supplement: S1 Table — (DOCX) [file pone.0209708.s002.docx]

**S1 Table** Comparison of mean TAC trough levels between -∆TAC_dose_ and +∆TAC_dose_ group during follow-up.

|  | Mean TAC trough levels | | *P*-value |
| --- | --- | --- | --- |
|  | -∆TAC_dose_ (n = 35) | +∆TAC_dose_ (n = 45) |  |
| Month 3 | 9.1 ± 1.2 | 8.7 ± 0.8 | 0.08 |
| Month 6 | 8.4 ± 0.7 | 8.6 ± 0.5 | 0.14 |
| Month 9 | 7.6 ± 0.7 | 7.8 ± 0.2 | 0.07 |
| Month 12 | 6.9 ± 0.9 | 7.1 ± 1.1 | 0.19 |
